# Comparison of Four Lymph Node Stage Methods for Predicting the Prognosis of Distal Cholangiocarcinoma Patients After Surgery

**DOI:** 10.3389/fonc.2021.779761

**Published:** 2021-12-03

**Authors:** Xiuyi Huang, Xiaoya Niu, Zhen You, Youlin Long, Fan Luo, Hui Ye

**Affiliations:** ^1^ Department of Biliary Surgery, West China Hospital, Sichuan University, Chengdu, China; ^2^ Chinese Evidence-Based Medicine Center, West China Hospital, Sichuan University, Chengdu, China

**Keywords:** distal cholangiocarcinoma (dCCA), log odds of positive lymph node (LODDS), lymph node stage, prognosis ability, modeling

## Abstract

**Background:**

The metastatic status of regional lymph nodes is an effective risk factor for the prognosis of distal cholangiocarcinoma (dCCA). But existing lymph node staging is not accurate enough and is susceptible to interference. This study aims to explore the predictive ability of the log odds of positive lymph nodes (LODDS) staging system of dCCA compared with existing lymph node staging systems.

**Methods:**

A total of 928 dCCA patients were selected from the Surveillance, Epidemiology, and End Results (SEER) database as the training cohort, and 207 dCCA patients from West China Hospital who underwent surgery were reviewed as the validation cohort. The least absolute shrinkage and selection operator (LASSO) and multivariate Cox regression were conducted to identify the most meaningful factors relevant to prognosis. The performance of four lymph node stage systems was compared by a model-based approach.

**Result:**

Age at diagnosis, pathological grade, American Joint Committee on Cancer (AJCC) tumor 7th T stage, tumor size, radiotherapy, chemotherapy, and lymph node stage system were independent prognostic factors. The model with the LODDS system had a better model fit with the highest C-index (0.679) and 1-/3-/5- area under the receiver operating characteristic curve (AUC) (0.739/0.671/0.658) as well as the lowest Akaike information criterion (AIC) (5,020.52). External validation results from 207 dCCA patients showed a C-index of 0.647 and 1-/3-/5-AUC of 0.740/0.683/0.589. Compared with the lymph node ratio (LNR), AJCC 8th N system, and 7th N system, the 5-year net reclassification improvement (NRI) of the LODDS system was 0.030 (95% CI: −0.079 to 0.147), 0.042 (95% CI: −0.062 to 0.139), and 0.040 (95% CI: −0.057 to 0.146), respectively. The integrated discrimination improvement (IDI) of LODDS improved compared with the LNR model (0.016; 95% CI: −0.001 to 0.036), AJCC 8th N system (0.020; 95% CI: 0.003–0.037), and AJCC 7th N system (0.019; 95% CI: 0.002–0.036). Decision curve analysis (DCA) also shows a greater net benefit of LODDS. In lymph node-negative patients, LODDS reveals a positive linear relationship with the hazard ratio (HR). The stage capacity of LODDS in a subgroup analysis stratified by examined lymph node number (ELNN) was consistent.

**Conclusions:**

The LODDS lymph node stage system has superior predictive performance as compared with the LNR, AJCC 7th, and 8th lymph node stage systems. Meanwhile, LODDS has a more detailed staging ability and good stability.

## Introduction

Distal cholangiocarcinoma (dCCA) is a malignant tumor located at the common bile duct, accounting for 20%–30% of all cholangiocarcinoma. Hitherto, the primary treatment for dCCA is radical surgical resection, such as pancreaticoduodenectomy and lymphadenectomy (1, 2). Due to the lower resection rate and the higher recurrence rate after surgery, the prognosis of dCCA is poor, and the 5-year survival rate for patients only ranges from 20% to 50% (3, 4). Many studies reported that the metastatic status of regional lymph nodes is a strong risk factor of prognosis (5–8). An accurate lymph node stage system is required to evaluate the tumor stage precisely and direct the appropriate postoperative adjuvant therapy for resectable dCCA after surgery.

The most commonly employed lymph node staging system so far is the American Joint Committee on Cancer (AJCC) tumor TNM staging system. In the AJCC 7th edition, the lymph node stage system of dCCA was based on the location of lymph node metastasis. The 8th edition lymph node stage was reclassified into N0 (no regional lymph node metastasis), N1 (1 to 3 regional lymph node metastasis), and N2 (≥4 regional lymph node metastasis) according to the regional positive lymph node number (PLNN). The 8th version also suggests that at least 12 lymph nodes need to be examined to ensure the accuracy of staging. However, dissection of more than 12 lymph nodes is a challenge sometimes due to the complexity of the surgical area and the skills of surgeons. Thus, a better lymph node staging system is required for the prognosis prediction of dCCA.

Lymph node ratio (LNR), which is the ratio of PLNN to examined lymph node number (ELNN), has been proposed as an indicator of lymph node staging. Several studies have confirmed that the LNR is a promising indicator of the prognosis of dCCA (4, 9–12). Log odds of positive lymph nodes (LODDS) is the log of the ratio of PLNN to negative lymph node number (NLNN). LODDS is theoretically more accurate than the LNR staging, especially in patients with negative lymph nodes. Good predictive ability in tumors has been shown in gallbladder cancer, hilar cholangiocarcinoma, colon cancer, gastric cancer, breast cancer, and thyroid cancers (13–18). However, the effect of LODDS has not been investigated in dCCA. Considering the latest lymph node stage method of dCCA conveying the significance of the number of lymph nodes toward prognosis, the LODDS may play a more important role in dCCA patients. In this study, we compare the performance of the LODDS staging system with three other lymph node staging systems (LNR system, AJCC 8th N staging system, and AJCC 7th N staging system) and explore more value of LODDS as a prognosis factor for dCCA after surgery.

## Method

### Study Design and Data Source

**Figure 1:**
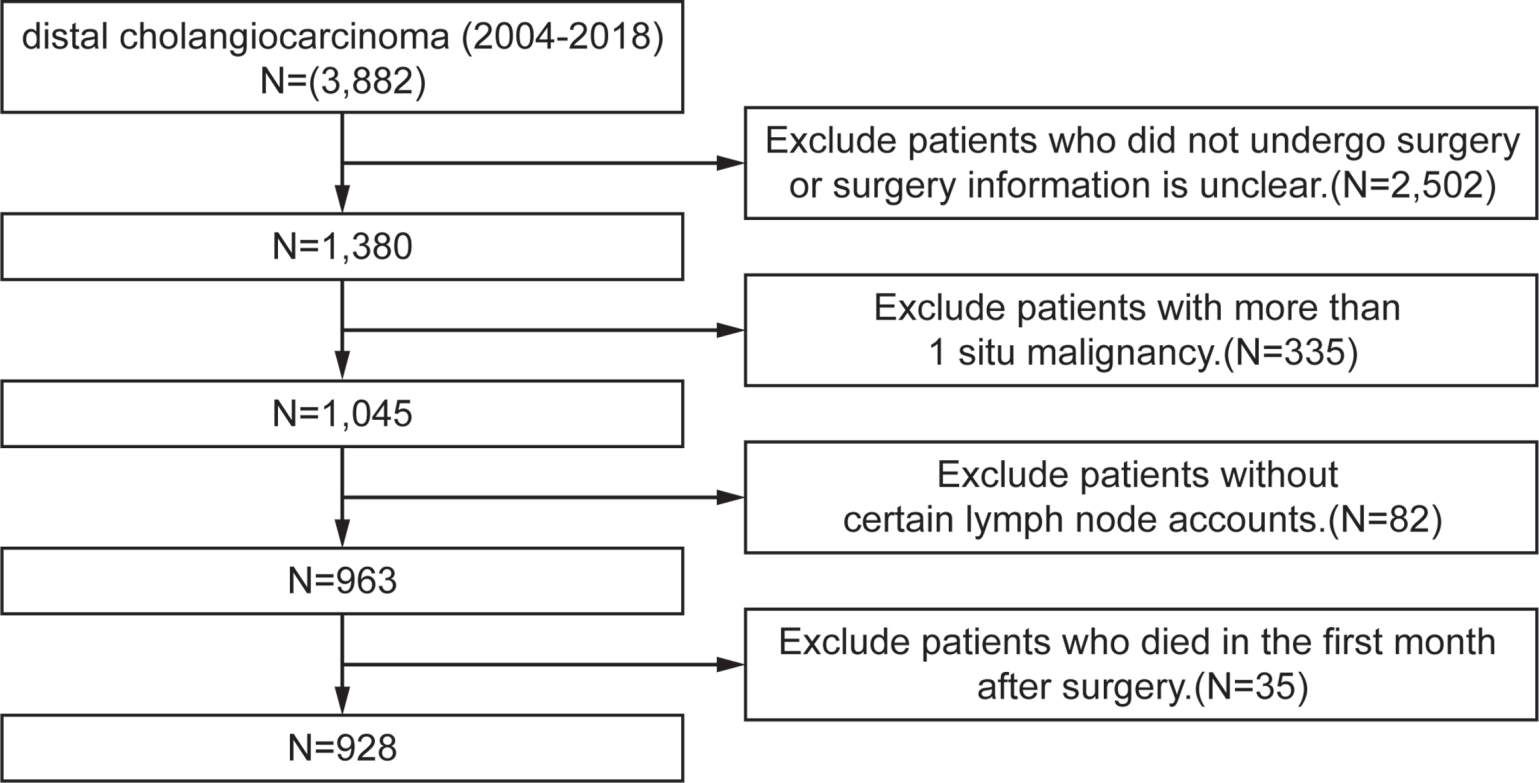
Flowchart of the data process for training set.

The Surveillance, Epidemiology, and End Results (SEER) database is an open-source clinical database. It collects cancer incidence data from population-based cancer registries covering approximately 34.6% of the U.S. population. The inclusion criteria are patients diagnosed with dCCA between 2004 and 2018 in the SEER database. The exclusion criteria are 1) patients who did not undergo surgery or surgery information is unclear; 2) patients with more than one *in situ* malignancy; 3) patients without certain lymph node accounts; and 4) patients who died in the first month after surgery. The process flowchart of the training cohort is shown in **Figure 1**. The primary outcome was overall survival (OS), which was defined as the time from diagnosis to death from any cause. The last follow-up date was November 31, 2020. The study was conducted following the Declaration of Helsinki. The Ethical Committee of The West China Hospital of Sichuan University has approved the research, and informed consent was obtained from all patients. The SEER database is an open-access database, and all patient information has been de-identified, so informed consent of the training set was waived.

### Handing of Variables

The variables we collected include the age at dCCA diagnosis, sex, race, histology, AJCC TNM stage, pathological grade, tumor size, ELNN, PLNN, NLNN, radiotherapy, and chemotherapy information, survival time, and vital status. The AJCC 8th lymph node stage was deduced by using the PLNN and metastasis region. Owing to lack of the information on the depth of bile duct infiltration in the database, the AJCC 7th T stage was still used in this study. LODDS is calculated by log [(0.05 + PLNN)/(0.05 + NLNN)], and the result of LODDS in this study ranges from −2.95 to 2.37. The LNR is the ratio of PLNN/ELNN, and the result ranges from 0 to 1.

### Handling of Missing Data

The missing data in the present study were about 14.5% including 70 cases in grade, 24 cases in TNM system, and 74 cases in tumor size. Some of the cases with more than one value are missing. By analyzing the relationship between the missing values and variables, we take that the data were missing at random. To fill the missing values, the multiple imputation method based on chained equations was performed 15 times. All the variables except survival time and status are included in the imputation procedure.

### Predictor Transformation and Selection

To increase utility and carry out further research, X-tile 3.6.1 software (Yale University School of Medicine, New Haven, CT, USA) was used to identify the optimal cutoff value of variables. The program calculates the cutoff value with significant differences by selecting the highest χ^2^ value and uses a standard log-rank test to calculate the p-value (19). The cutoff values of age are 78 years; the tumor size was categorized as <20, 20 to 34, and ≥ 34 mm. LODDS was divided into three categories: LODDS1–3, <−2.0, −2.0 to −0.4, and ≥−0.4. The LNR was stratified into LNR1–3: <0.1, 0.1 to 0.3, and ≥0.3. The rest of the category variables were classified according to the classification criteria of the SEER database.

The least absolute shrinkage and selection operator (LASSO) *via* 10-fold cross-validation was conducted firstly. Twelve variables were selected with the lambda value that produces the minimum mean cross-validated error: age at diagnosis, race, pathological grade, AJCC 7th T stage, LODDS, tumor size, NLNN, radiotherapy, and chemotherapy. Then the selected variables above, as well as the AJCC 7th N stage, AJCC 8th N stage, and LNR, were incorporated into multivariable Cox regression.

### Model Construction, Comparison, and Validation

Cox proportional hazard models of four N staging systems (LODDS system, LNR system, AJCC 8th N staging system, and AJCC 7th N staging system) based on the variables screened above are constructed. The proportionality of hazards in Cox models was tested based on the Schoenfeld residuals, and chemotherapy was considered as a time-dependent covariable. Thus, a step function was used to estimate the hazard ratio (HR) according to the median OS, and the observation period was split into ≤26 and >26 months. Akaike information criterion (AIC), C-index, and the area under the receiver operating characteristic curve (AUC) of 1/3/5 years of four models were compared. The net reclassification improvement (NRI) and the integrated discrimination improvement (IDI) were calculated to cross-sectionally compare the predictive power of LODDS with other lymph node staging systems. The levels of year risk are derived from the event rate (20). Decision curve analysis (DCA) was used to compare the clinical availability and utility of four lymph node stage models. Finally, external validation of the model was carried out by using patients filtered at the West China Hospital of Sichuan University.

### Statistical Analysis

The group differences of categorical and continuous variables were determined by the chi-square test and Kruskal–Wallis H test, respectively. The Cox proportional hazards regression model of four lymph node stages was constructed, the survival curve of each model was depicted by the Kaplan–Meier analysis, and the differences were tested by a log-rank method. Statistical analyses were carried out with R software version 4.0.4. A p-value <0.05 was regarded as significant.

## Results

### Demographic and Clinical Characteristics

928 patients in total diagnosed with dCCA between 2004 and 2018 from the SEER database were selected as the training cohort. 207 patients diagnosed with dCCA at the West China Hospital of Sichuan University from 2009 to 2018 were collected as the validation cohort. The baseline characteristics of the training cohort and validation cohort are summarized in **Table S1**. For the training set, the median follow-up time was 45 months; the median OS was 26 months; and 3- and 5-year survival was 37.7% and 26%, respectively. The median age was 67 years (interquartile range (IQR): 59.3–73.0); more patients were male (66.6%); the median size of tumors was 22 mm (IQR: 15.0–30.0); more than half of patients received chemotherapy; and 31.2% of patients received radiation therapy. The median and mean values of ELNN are 14.0 and 15.0, respectively; the median and mean values of PLNN are 1 and 1.7, respectively.

### Prognostic Factors for Distal Cholangiocarcinoma Patients

Variables were then subjected to multivariate Cox regression such as age at diagnosis, sex, race, pathological grade, AJCC 7th T stage, LODDS, LNR, tumor size, ELNN, PLNN, radiotherapy, and chemotherapy filtered by LASSO regression. The HRs of the variables above with corresponding 95% CIs are presented in **Table 1**. Age at diagnosis, pathological grade, tumor size, chemotherapy, and lymph node stage system were closely associated with the OS of dCCA (p < 0.05). Though the p-value of radiotherapy is not significant in multivariate Cox regression (HR = 0.92, p = 0.463), it was still regarded as a predictor since relevant studies have shown the improved prognosis of dCCA with radiotherapy (21–24). Finally, seven variables were ascertained to construct the model: age at diagnosis, pathological grade, AJCC 7th T stage, tumor size, radiotherapy, chemotherapy, and lymph node stage system. The variance inflation factor value for all variables is between 1 and 5, which means moderate multicollinearity between variables in the model and does not need to be adjusted (25).

**Table 1:** Multivariate Cox regression analysis of factors associated with overall survival.

Variables		Multivariate analysis		
		HR	95% CI	p-Value
Age, year	≤78	Reference		
	>78	1.55	1.17–2.05	0.002**
Race	White	Reference		
	Black	0.85	0.61–1.2	0.356
	Other	0.89	0.71–1.11	0.305
Grade	Well, I	Reference		
	Moderately, II	1.14	0.84–1.54	0.398
	Poorly, III	1.43	1.05–1.95	0.025*
7th T stage	T1	Reference		
	T2	1.39	0.96–2.03	0.085
	T3	2.04	1.42–2.94	<0.001***
	T4	2.04	1.23–3.39	0.006**
7th N stage	N0	Reference		
	N1	2.20	0.66–7.29	0.198
8th N stage	N0	Reference		
	N1	0.43	0.13–1.51	0.190
	N2	0.57	0.15–2.17	0.414
LODDS	LODDS 1	Reference		
	LODDS 2	1.70	1.21–2.38	0.002**
	LODDS 3	4.32	2.16–8.63	<0.001***
LNR	LNR 1	Reference		
	LNR 2	1.04	0.77–1.41	0.793
	LNR 3	0.51	0.26–1.01	0.054
Tumor size (cm)	≤20	Reference		
	20–34	1.07	0.87–1.31	0.519
	>34	1.67	1.32–2.12	<0.001***
NLNN	—	0.99	0.97–1	0.026*
Chemotherapy	None/unknown	Reference		
	Yes	0.71	0.57–0.88	0.002**
Radiotherapy	None/unknown	Reference		
	Yes	0.92	0.74–1.15	0.463

HR, hazard ratio; T, tumor; N, node; LODDS, log odds of positive lymph nodes; LNR, positive lymph node ratio; PLNN, positive lymph nodes number; NLNN, negative lymph node number.

*p-Values <0.05.

**p-Values <0.01.

***p-Values <0.001.

### Comparison of Four Lymph Node Staging Systems and Validation

**Figure 2:**
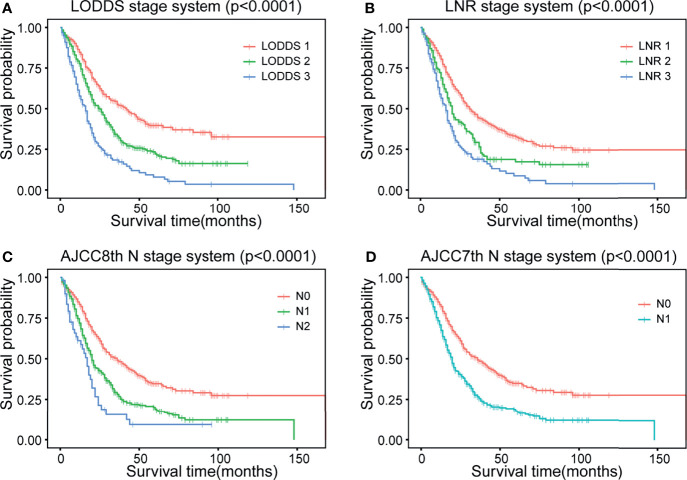
Kaplan–Meier curves of four different lymph node staging systems. **(A)** LODDS system, **(B)** LNR system, **(C)** AJCC 8th N stage, and **(D)** AJCC 7th N stage. LODDS, log odds of positive lymph nodes; LODDS1, <−2.0; LODDS2, −2.0 to −0.4; LODDS3, ≥−0.4; LNR, lymph node ratio; LNR1, <0.1; LNR2, 0.1 to 0.3; LNR3, ≥0.3; AJCC, American Joint Committee on Cancer. AJCC 8th: N0, no regional lymph node metastasis; N1, 1 to 3 regional lymph node metastasis; N2, ≥4 regional lymph node metastasis. AJCC 7th: N0, no regional lymph node metastasis; N1, positive regional lymph nodes. OS, overall survival.

**Figure 3:**
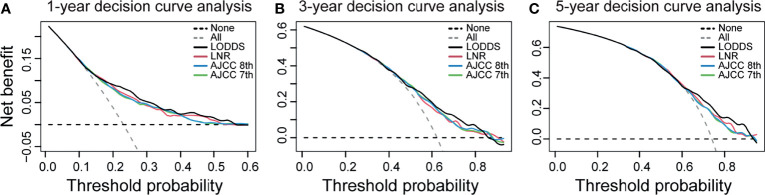
Decision curve analysis for the LODDS system, LNR system, AJCC 8th N stage, and AJCC 7th N stage. **(A)** 1-year decision curve analysis. **(B)** 3-year decision curve analysis. **(C)** 5-year decision curve analysis. The x-axis indicates net benefit when all patients are considered as not having the outcome, and the y-axis indicates net benefit when all patients are considered as having the outcome. The LODDS has the highest net benefit over the other three lymph node models mostly. LODDS, log odds of positive lymph nodes; LNR, lymph node ratio; AJCC, American Joint Committee on Cancer.

The strata method of four N staging systems in the Kaplan–Meier survival were all statistically significant for OS of dCCA (p < 0.0001, log-rank; **Figure 2**), indicating that the four lymph node stage systems are all well predictive of prognosis for dCCA. **Table 2** lists and compares the model parameters of four models. The C-index for the LODDS, LNR, AJCC 8th, and 7th N systems were 0.679, 0.672, 0.667, and 0.666, respectively. The AIC values of four lymph node stages were 5,020.52, 5,036.77, 5,041.48, and 5,044.56. The 1-year AUC values for four lymph node stages were 0.739, 0.728, 0.718, and 0.715. The 3-year AUC values for four lymph node stages were 0.671, 0.655, 0.664, and 0.665. The 5-year AUC values for four lymph node stages were 0.658, 0.653, 0.643, and 0.644. The LODDS system had the best model fit with the highest C-index and 1-/3-/5-AUC as well as the lowest AIC. For this reason, LODDS was considered the most accurate way of lymph node staging for the prognosis of dCCA patients. NRI and IDI were calculated to measure the reclassification improvements of LODDS (**Table 2**). The 5-year NRI values of LODDS vs. LNR, AJCC 8th, and AJCC 7th N systems were 0.030 (95% CI: −0.079 to 0.147), 0.042 (95% CI: −0.062 to 0.139), and 0.040 (95% CI: −0.057 to 0.146). The 5-year IDI values of LODDS vs. LNR, AJCC 8th, and 7th N systems were 0.016 (95% CI: −0.001 to 0.036), 0.020 (95% CI: 0.003–0.037), and 0.019 (95% CI: 0.002–0.036), respectively. All values of NRI and IDI are greater than zero, which implies that LODDS can better differentiate between high- and low-risk dCCA patients after surgery, especially for low-risk patients who are classified as high risk by the original staging approach. The DCA curve shows that the LODDS system has the highest net benefits among the four lymph node stage systems, which indicates better performance in prognostic predictions for dCCA (**Figure 3**).

**Table 2:** The comparison of four lymph node stage system.

System	LODDS	LNR	8th N stage^†^	7th N stage^†^
AIC	5,020.52	5,036.77	5,041.48	5,044.56
AUC (1-year)	0.739	0.728	0.718	0.715
AUC (3-year)	0.671	0.655	0.664	0.665
AUC (5-year)	0.658	0.653	0.643	0.644
C-index	0.679	0.672	0.667	0.666
**LODDS model performance compared with other models**				
NRI (5-year)	—	0.030 (−0.079 to 0.147)	0.042 (−0.062 to 0.139)	0.040 (−0.057 to 0.146)
NRI (events)	—	−0.027 (−0.077 to 0.024)	−0.024 (−0.065 to 0.026)	−0.024 (−0.061 to 0.024)
NRI (non-events)	—	0.057 (−0.053 to 0.176)	0.064 (−0.034 to 0.153)	0.064 (−0.034 to 0.164)
IDI (5-year)	—	0.016 (−0.001 to 0.036)	0.020 (0.003 to 0.037)	0.019 (0.002 to 0.036)

LODDS, log odds of positive lymph nodes; LNR, positive lymph node ratio; AIC, Akaike information criterion; AUC, the area under the receiver operating characteristic curve; NRI, net reclassification improvement; IDI, integrated discrimination improvement.

^†^American Joint Committee on Cancer TNM staging system.

External validation results from 207 dCCA patients showed a C-index of 0.6476 and 1-/3-/5-AUC of 0.740/0.683/0.589, respectively. The results indicate a good predictive ability of LODDS in the Asian population. And the accurate predictive capability of LODDS also has been proved in DCA curve (**Figure 3**).

### Relationship Between the Number of Lymph Nodes and Staging

**Figure 4:**
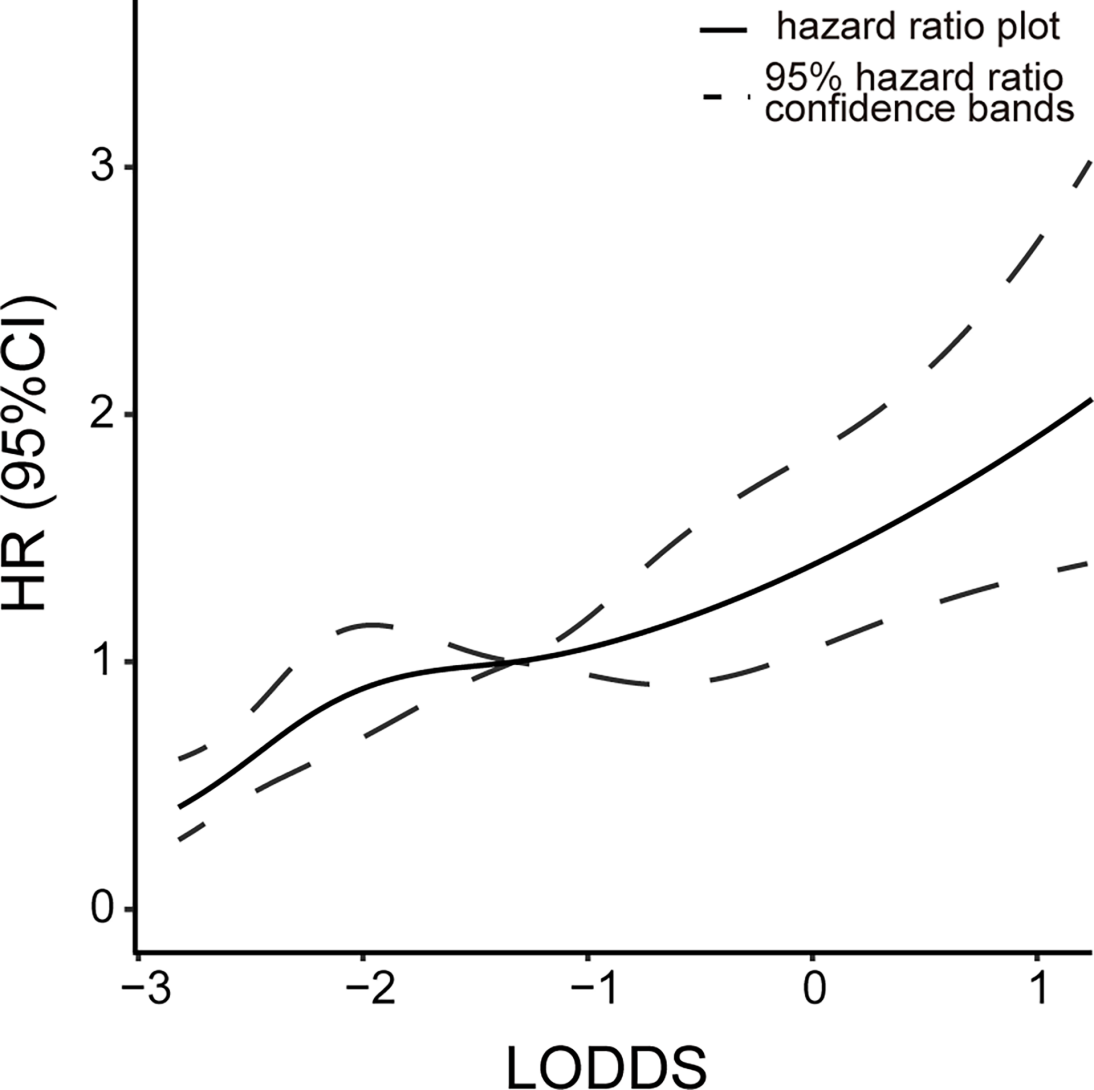
Relationship between the risk of death and LODDS value at LNR = 0. Continuous line, hazard ratio plot; dotted lines, 95% hazard ratio confidence bands. LODDS, log odds of positive lymph nodes; LNR, lymph node ratio.

When all tested lymph nodes are negative, the LNR value is equal to 0 and the N stage of both AJCC 7th and 8th is N0. But the LODDS value still varies based on the number of lymph nodes detected. We explored the correlations between HR and LODDS when PLNN = 0. **Figure 4** shows a positive linear relationship between LODDS and HR. This suggests that the ELNN in lymph node-negative patients is prognostically relevant and that LODDS has the ability to predict risk in this condition.

**Figure 5:**
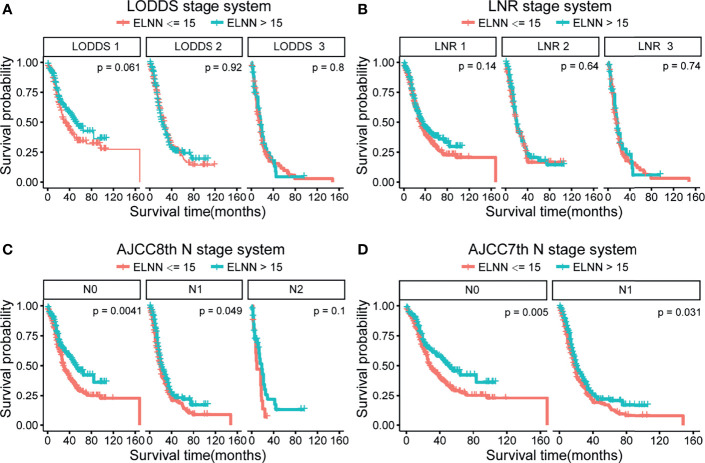
Kaplan–Meier survival curve according to N stage for the four lymph node staging systems stratified by ELNN=15. **(A)** LODDS system, **(B)** LNR system, **(C)** AJCC 8th N stage, and **(D)** AJCC 7th N stage. LODDS, log odds of positive lymph nodes; ELNN, examined lymph node number; LNR, lymph node ratio; AJCC, American Joint Committee on Cancer; OS, overall survival.

To investigate the impact of ELNN on prognosis and staging accuracy, the effect of ELNN controlling for the risk of death was evaluated in **Table S2**. Patients’ outcomes significantly improved when the ELNN is more than 15. We additionally performed subgroup-stratified analyses, identifying the stage accuracy of four systems stratified by ELNN. The cutoff value of ELNN is 15. The Kaplan–Meier survival shows that there is a significant difference in the prognosis of AJCC 7th and AJCC 8th N stages stratified by ELNN = 15 (**Figure 5**). But the p-value was not significant in all strata of the LODDS and LNR staging systems. This suggests that AJCC 7th and 8th N stage methods tend to be influenced by the ELNN while the LODDS and LNR are less disturbed by ELNN. Thus, the LODDS and LNR perform better in improving staging accuracy in cases of an inadequate number of lymph nodes detected.

### Chemotherapy and Prognosis

When the data were modeled as a step function by adjusting the covariates, we discover that there was a negative association between chemotherapy and mortality until approximately 12 months after surgery (HR = 0.41, 95% CI: 0.30–0.56, p < 0.001), after which there was a positive association between chemotherapy exposure and mortality (**Table S3**). This phenomenon gives us an unexpected discovery that chemotherapy may be associated with improved early prognosis.

## Discussion

The importance of lymph node metastasis status when assessing disease prognosis is necessary to be taken into account. Thus, an appropriate method for lymph node staging is helpful when guiding the therapy of dCCA after surgery. In the 7th edition of the AJCC TNM staging system, the N staging is mainly distinguished by the presence of regional lymph node metastasis. Several studies have confirmed that the number of regional PLNN can reflect the effect of lymph node metastasis on the prognosis of dCCA better when compared with the presence of lymph node metastases alone (26–30). The other two retrospective studies also found that patients with more than 11 lymph nodes removed had a better prognosis (31, 32). Besides, a large multicenter cohort studied by Kang et al. concluded that PLNN detection rates become stabilized when the total number of dissected lymph nodes was more than 12 (33). Thus, the 8th version adopted the regional PLNN as the N stage. To ensure the staging accuracy, the recommended minimum number of lymph nodes to be detected is 12 (34). In our study, the association with prognosis was not significant when the cutoff value of ELNN was 12 (HR = 0.92, 95% CI: 0.777–1.090, p = 0.335). However, with more than 15 lymph nodes, the dissection prognosis was improved significantly. One reason for the discrepancy may be that the SEER database lacks information for excluding high-risk patients, such as marginal status.

Although examining an adequate number of lymph nodes can help stage precisely, we also need to consider the accuracy of staging when the ELNN is inadequate. The LNR staging system combined with ELNN and PLNN compensates for the impact of insufficient ELNN on staging. A meta-analysis found that LNR = 0.2 is the ideal cutoff value for risk stratification of dCCA patients with radical resection (4), while the optimal cutoff value based on prognosis was between 0.45 and 0.17 (11, 12, 29, 30). Similar cutoff values were obtained in our study by using X-tile software: LNR1-3: <0.1, 0.1 to 0.3, and ≥0.3. However, the LNR is also incomplete. The LNR value becomes 0 or 1 when PLNN is 0 or all examined lymph nodes are positive, which lacks the corresponding discriminatory ability (35). LODDS is the log of the ratio of PLNN to NLNN. It could stage the lymph node condition when the PLNN is 0 or fully positive as well. The linear relationship between LODDS and HR at PLNN = 0 depicted in our study suggests that LODDS has good discriminating power in lymph node-negative patients. The value of LODDS in the fully positive lymph node patients is not calculated due to few samples. To evaluate the predictive power of different N stages when considering ELNN, subgroup analysis was performed to compare survival curves of four lymph node stages. Since the detection of 12 lymph nodes suggested by previous articles does not significantly impact the prognosis in our study, we choose 15 as the cutoff value of ELNN. Both the LODDS and LNR models are unaffected by staging migration arising from the number of lymph nodes detected. This demonstrates the advantages of LODDS staging from another aspect. However, since the SEER database lacks other information on relevant risk factors, it may affect the results.

To identify the prognostic value of LODDS, this study calculated the values of C-index, AIC, and AUC of the four lymph node stage models, and the LODDS presented the best performance overall. The universality of the conclusion was verified by using data from our medical center. The result showed the superiority of LODDS as a lymph node stage system and also performs a good predictive ability in the Asian population. Eventually, the NRI, IDI values, and DCA curves exhibited improvement compared with the AJCC 8th staging system, AJCC 7th N staging system, and LNR staging system. This further confirmed the predictive accuracy of LODDS.

In addition, we observed a time-varying correlation between chemotherapy and time-related hazard. Chemotherapy significantly decreased the risk of mortality in the first year after surgery but has no significant improvement in prognosis beyond 1 year, which may indicate that chemotherapy improves the early prognosis of dCCA patients after surgery. Besides, we take radiotherapy as a predictor based on clinical experience. The conduction of radiotherapy for dCCA has not come to a global agreement. Several studies have revealed that postoperative chemoradiotherapy improves the prognosis of patients significantly, especially for patients with R1 resection and regional LN metastases. But the effect is not remarkable when chemotherapy or radiotherapy is given alone (21–24, 36–38). In our study, radiotherapy was not significant for OS improvement but benefit prognosis when excluding patients with negative regional lymph node metastasis (HR = 0.725, p = 0.006). We can reasonably assume that radiotherapy improves the prognosis of high-risk patients from previous studies. However, large prospective randomized controlled trials are required for exploring the effect of adjuvant therapy on dCCA. Meanwhile, jaundice, high fibrinogen level, and alcohol consumption could be associated with a poor prognosis of dCCA after pancreatoduodenectomy (39). But we could not take these factors into account in the analysis due to a lack of information in the SEER database. This might limit our model’s performance. To the extent of our knowledge, this study is the first one that investigates the predictive value of the LODDS lymph node staging system in dCCA. The results demonstrated that LODDS staging outperformed the AJCC staging and LNR staging. Nevertheless, some shortcomings still exist in this study. First, the data in the SEER database are retrospective and therefore was exposed to selection bias. Second, some other vital information related to the tumor, like marginal status, tumor markers, AJCC 8th T stage, and jaundice, is not recorded in the SEER database, which may affect the accuracy of the prediction model. Third, the cutoff values of LODDS and tumor size are calculated from the log-rank test, and their validity needs to be further confirmed in large samples of clinical practice.

## Conclusion

For patients with dCCA, the LODDS lymph node stage system seems to have a superior ability to predict survival compared with the AJCC staging system and LNR systems. Particularly, it compensates for the migration of the AJCC lymph node stage system better when the number of lymph nodes examined is low, as well as the accurate stage of lymph node-negative patients. Hence, the LODDS lymph node stage may be a promising predictor.

## Data Availability Statement

The original contributions presented in the study are included in the article/**Supplementary Material**. Further inquiries can be directed to the corresponding author.

## Ethics Statement

The studies involving human participants were reviewed and approved by the Ethics Committee on Biomedical Research, West China Hospital of Sichuan University. The patients/participants provided their written informed consent to participate in this study.

## Author Contributions

XH: conceptualization, methodology, survey, data analysis, and writing—original draft. ZY: methodology, survey, and data analysis. XN: survey and data collection. YL: methodology and data analysis. FL: data collection. HY: writing—review and editing, and supervision. All authors contributed to the article and approved the submitted version.

## Conflict of Interest

The authors declare that the research was conducted in the absence of any commercial or financial relationships that could be construed as a potential conflict of interest.

## Publisher’s Note

All claims expressed in this article are solely those of the authors and do not necessarily represent those of their affiliated organizations, or those of the publisher, the editors and the reviewers. Any product that may be evaluated in this article, or claim that may be made by its manufacturer, is not guaranteed or endorsed by the publisher.
