# Supplementary material for: Comparison of Four Lymph Node Stage Methods for Predicting the Prognosis of Distal Cholangiocarcinoma Patients After Surgery
Source: Front Oncol. 2021 Dec 3;11:779761. doi: 10.3389/fonc.2021.779761 (PMC8678514; doi:10.3389/fonc.2021.779761)
Supplement: Supplementary file 1 [file Table_1.pdf]

Table S1. Demographic and tumor data of all dCCA patients after surgery.

Characters	All subjects (n=1135),n(%)	Training cohort (n=928),n(%)	Validation cohort (n=207),n(%)	P value
Age(year) (median [IQR])	65.00 [58.00, 72.00]	67.00 [59.25, 73.00]	61.00 [53.00, 66.00]	<0.001***
Race				<0.001***
White	692 (61.0)	692 (74.6)	0 (0.0)	<0.001***
Black	72 (6.3)	72 (7.8)	0 (0.0)	
Other	371 (32.7)	164 (17.7)	207 (100.0)	
Gender				0.044*
Male	740 (65.2)	618 (66.6)	122 (58.9)	
Female	395 (34.8)	310 (33.4)	85 (41.1)	
Histology				0.206
Adenocarcinoma	1067 (94.0)	868 (93.5)	199 (96.1)	
Other	68 (6.0)	60 (6.5)	8 (3.9)	
Grade				<0.001***
Well, I	106 (9.3)	100 (10.8)	6 (2.9)	
Moderately II	542 (47.8)	423 (45.6)	119 (57.5)	
Poorly III	417 (36.7)	335 (36.1)	82 (39.6)	
Missing	70 (6.2)	70 (7.5)	0 (0.0)	
†7th TNM stage				<0.001***
IA	87 (7.7)	76 (8.2)	11 (5.3)	
IB	154 (13.6)	115 (12.4)	39 (18.8)	
IIA	281 (24.8)	187 (20.2)	94 (45.4)	
IIB	482 (42.5)	428 (46.1)	54 (26.1)	
III	65 (5.7)	63 (6.8)	2 (1.0)	
IV	42 (3.7)	35 (3.8)	7 (3.4)	
Missing	24 (2.1)	24 (2.6)	0 (0.0)	
7th T stage				0.001**
T1	114 (10.0)	103 (11.1)	11 (5.3)	
T2	304 (26.8)	254 (27.4)	50 (24.2)	
T3	657 (57.9)	515 (55.5)	142 (68.6)	
T4	40 (3.5)	36 (3.9)	4 (1.9)	
Missing	20 (1.8)	20 (2.2)	0 (0.0)	
7th M stage				0.247
M0	1081 (95.2)	881 (94.9)	200 (96.6)	
M1	42 (3.7)	35 (3.8)	7 (3.4)	
Missing	12 (1.1)	12 (1.3)	0 (0.0)	
7th N stage				<0.001***
N0	598 (52.7)	451 (48.6)	147 (71.0)	
N1	537 (47.3)	477 (51.4)	60 (29.0)	
8th N stage				<0.001***
N0	599 (52.8)	452 (48.7)	147 (71.0)	
N1	470 (41.4)	425 (45.8)	45 (21.7)	
N2	66 (5.8)	51 (5.5)	15 (7.2)	
LNR	0.00 [0.00, 0.17]	0.04 [0.00, 0.17]	0.00 [0.00, 0.13]	<0.001***

(median [IQR])				
LODDS (median [IQR])	-1.33 [-2.26, -0.68]	-1.33 [-2.34, -0.67]	-1.79 [-2.08, -0.79]	0.914
Tumor size (cm) (median [IQR])	22.00 [15.00, 30.00]	22.00 [15.00, 30.00]	20.00 [15.00, 30.00]	0.985
Missing	74 (6.5)	74 (8.0)	0 (0.0)	
ELNN (median [IQR])	12.00 [5.00, 20.00]	14.00 [7.00, 22.00]	5.00 [2.00, 8.00]	<0.001***
PLNN (median [IQR])	0.00 [0.00, 2.00]	1.00 [0.00, 2.00]	0.00 [0.00, 1.00]	<0.001***
Chemotherapy				<0.001***
Yes	605 (53.3)	548 (59.1)	57 (27.5)	
None/unknown	530 (46.7)	380 (40.9)	150 (72.5)	
Radiotherapy				<0.001***
Yes	302 (26.6)	290 (31.2)	12 (5.8)	
None/unknown	833 (73.4)	638 (68.8)	195 (94.2)	

*, *P* values <0.05; **; *P* values <0.01; ***, *P* values <0.001. dCCA, Distal cholangiocarcinoma; IQR, interquartile range; TNM, tumor-node-metastasis; T, tumor; N, node; M, metastasis; LNR, positive lymph node ratio; LODDS, log odds of positive lymph nodes; ELNN, examined lymph node number; PLNN, positive lymph node number. †, The total number of missing data of 7th TNM stage is 24, including 7 patients missing both T and M stage.

Table S2. Death risk of dCCA patients according to the value of ELNN.

Group	HR	HR.95L	HR.95H	P value
ELNN > 2	0.808	0.618	1.058	0.121
ELNN > 3	0.809	0.643	1.018	0.071
ELNN > 4	0.817	0.661	1.01	0.062
ELNN > 5	0.803	0.659	0.979	0.030*
ELNN > 6	0.809	0.669	0.979	0.029*
ELNN > 7	0.895	0.745	1.075	0.234
ELNN > 8	0.885	0.740	1.059	0.182
ELNN > 9	0.927	0.778	1.106	0.402
ELNN > 10	0.938	0.789	1.115	0.469
ELNN > 11	0.902	0.76	1.069	0.234
ELNN > 12	0.92	0.777	1.090	0.335
ELNN > 13	0.886	0.748	1.050	0.162
ELNN > 14	0.843	0.711	1.000	0.050
ELNN > 15	0.811	0.683	0.964	0.017*
ELNN > 16	0.837	0.703	0.996	0.045*
ELNN > 17	0.808	0.676	0.966	0.019*
ELNN > 18	0.774	0.643	0.931	0.006**
ELNN > 19	0.804	0.666	0.971	0.023*
ELNN > 20	0.798	0.655	0.972	0.025*

dCCA, distal cholangiocarcinoma; ELNN, examined lymph node number; HR, hazard ratio.

Table S3. Estimated Hazard ratio of chemotherapy related to postoperative time.

Month after surgery	HR of chemotherapy	HR.95L	HR.95H	P value
≤ 12	0.41	0.30	0.56	<0.001***
12-24	0.99	0.71	1.37	0.947
24-36	1.26	0.78	2.04	0.348
36-48	1.57	0.77	3.24	0.218
48-60	0.57	0.21	1.54	0.270
> 60	0.64	0.23	1.77	0.386

HR: hazard ratio.
